# Removal of Radioactive Cesium Using Prussian Blue Magnetic Nanoparticles

**DOI:** 10.3390/nano4040894

**Published:** 2014-11-28

**Authors:** Sung-Chan Jang, Sang-Bum Hong, Hee-Man Yang, Kune-Woo Lee, Jei-Kwon Moon, Bum-Kyoung Seo, Yun Suk Huh, Changhyun Roh

**Affiliations:** 1 Decontamination and Decommissioning Research Division, Korea Atomic Energy Research Institute (KAERI), 989-111 Daedukdaero Yuseong, Daejeon 305-353, Korea; E-Mails: jsc@kaeri.re.kr (S.-C.J.); sbhong@kaeri.re.kr (S.-B.H.); hmyang@kaeri.re.kr (H.-M.Y.); nkwlee@kaeri.re.kr (K.-W.L.); njkmoon@kaeri.re.kr (J.-K.M.); 2 Department of Biological Engineering, Biohybrid Systems Research Center (BSRC), Inha University, Incheon 402-751, Korea; 3 Biotechnology Research Division, Advanced Radiation Technology Institute (ARTI), Korea Atomic Energy Research Institute (KAERI), 1266, Sinjeong-dong, Jeongeup, Jeonbuk 580-185, Korea

**Keywords:** radioactive cesium (^137^Cs), magnetic nanoparticle, remediation

## Abstract

Radioactive cesium (^137^Cs) has inevitably become a human concern due to exposure from nuclear power plants and nuclear accident releases. Many efforts have been focused on removing cesium and the remediation of the contaminated environment. In this study, we elucidated the ability of Prussian blue-coated magnetic nanoparticles to eliminate cesium from radioactive contaminated waste. Thus, the obtained Prussian blue-coated magnetic nanoparticles were then characterized and examined for their physical and radioactive cesium adsorption properties. This Prussian blue-coated magnetic nanoparticle-based cesium magnetic sorbent can offer great potential for use in* in situ* remediation.

## 1. Introduction

Radioactive cesium (^137^Cs) removal has become an emerging issue after the Fukushima Daiichi Nuclear Power Plant Disaster [1,2,3,4]. Thus, radioactive cesium due to its high radioactivity and relatively long half-life time (30.2 years) is a significant component of nuclear wastes and nuclear fallout [5,6,7]. The cesium is water-soluble and behaves similarly to potassium and sodium in the biological behavior profile. Additionally, a high dose of radioactive ^137^Cs can induce medullar dystrophy and disorders of reproductive function. Moreover, it can cause a number of negative health effects, including carcinoma of the liver, kidney, bladder, renal functions, cardiovascular disease and gastrointestinal distress [8,9,10]. Prussian blue, known as ferric(III) hexacyanoferrate(II), is a pigment of dark blue color and was one of the first synthetic dyes. Prussian blue is a complex composed of Fe_4_[Fe(CN)_6_]_3_·*X*H_2_O (*X* = 14–16) with a cubic face-centered lattice structure [11]. Prussian blue is known as a low-cost adsorbent, which has a high selectivity for cesium, including a high stability, high conductivity, biocompatibility, size controllability and easy surface functionalization for decomposition [12]. Furthermore, Prussian blue is a typical FDA-approved drug used in clinics for the safe treatment of radioactive exposure [13,14,15]. Additionally, magnetic nanoparticles can be easily separated from a treated solution by the simple application of either a permanent or electro magnet and have made it possible to utilize several advantages of the sorbent without the typical difficulty associated with the separation of nanoparticles from solution [16,17]. In this study, modified Prussian blue, including γ-Fe_2_O_3_ magnetic nanoparticles, was synthesized and characterized by Fourier-transform infrared (FTIR), X-ray diffraction (XRD), transmission electron microscopy (TEM) and scanning electron microscopy (SEM).

## 2. Results and Discussion

### 2.1. Synthesis of Prussian Blue-Coated Magnetic Nanoparticles

**Figure 1:**
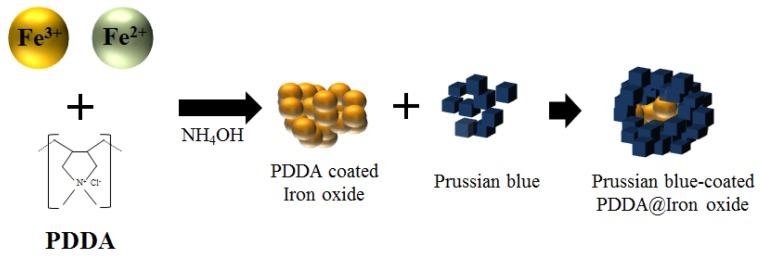
Synthetic procedures for the Prussian blue-coated magnetic nanoparticles. PDDA, poly(diallyldimethylammonium chloride).

To coat soluble Prussian blue on the surface of a poly(diallyldimethylammonium chloride) (PDDA) coated iron oxide nanoparticle, it is possible to react the hexacyanoferrate(II) ions with the ferric ions in the presence of PDDA@Iron oxide nanoparticles. In this case, the PDDA@Iron oxide nanoparticles can act as nucleation sites for the precipitated Prussian blue, resulting in the coating of the Prussian blue on to the PDDA@Iron oxide nanoparticle surface. Synthetic procedures for Prussian blue-coated magnetic nanoparticles are presented in Figure 1. The scheme represents that the adsorbents are composed largely of negatively-charged Prussian blue with a smaller amount of positively-charged PDDA@Iron oxide and that the surface area of the positively-charged PDDA@Iron oxide nanoparticles is entirely covered with negatively-charged Prussian blue.

**Figure 2:**
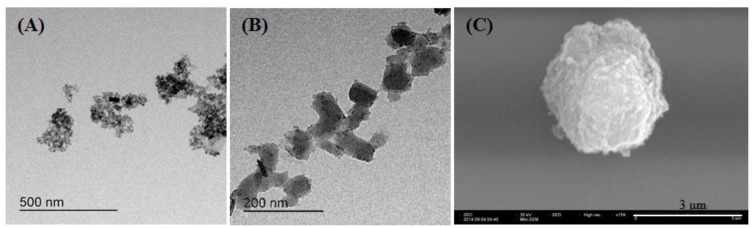
Electron microscope images. (**A**) Transmission electron microscopy (TEM) image of PDDA-coated iron oxide; (**B**) TEM image of Prussian blue; (**C**) Scanning electron microscopy (SEM) image of Prussian blue-coated PDDA@Iron oxide.

Additionally, the formation of the nanoparticles was evaluated by using TEM and SEM. Finally, the electron micrographs revealed that the average particle sizes of the Prussian blue-coated PDDA@Iron oxide were less than 3 ìm (Figure 2). The size of Prussian blue-coated PDDA@Iron oxide by DLS was less than 500 nm. Additionally, the zeta potentials of Prussian blue and PDDA@Iron oxide were −40 mV and +35 mV, respectively.

**Figure 3:**
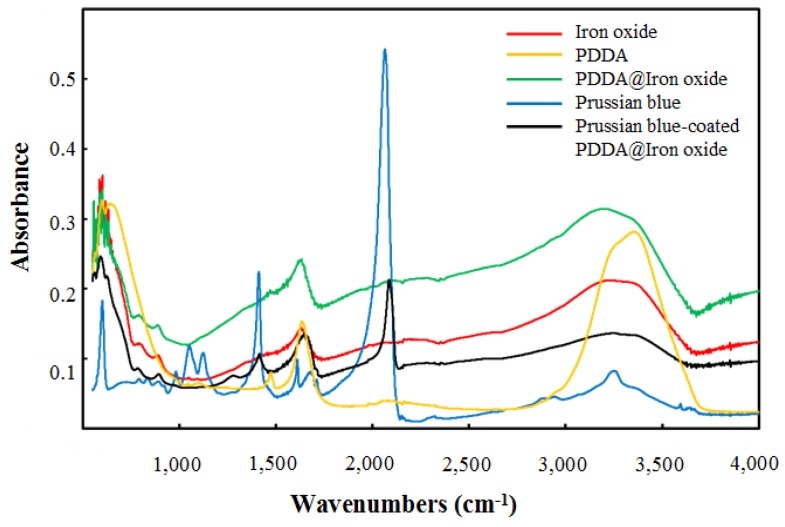
Fourier-transform infrared (FTIR) spectra of the synthesized materials.

**Figure 4:**
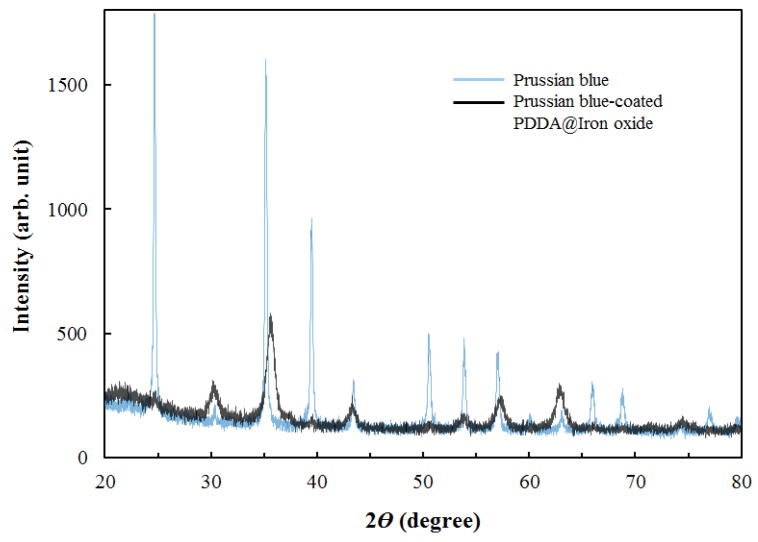
X-ray diffraction (XRD) peaks of Prussian blue and Prussian blue-coated PDDA@Iron oxide.

In Figure 3, the FTIR spectra of the materials were examined. The peaks of bands at 1614 cm^−1^ and 3379 cm^−1^ represent the existence of iron oxide. A band at 1472 cm^−1^ indicated the existence of PDDA in the PDDA@Iron oxide. Prussian blue had an intense band at ~2076 cm^−1^. This spectrum was not altered when the Prussian blue was combined with PDDA@Iron oxide and was identical to its Prussian blue-coated PDDA@Iron oxide spectrum. Subsequently, the crystal structure of Prussian blue-coated PDDA@Iron oxide was examined. Figure 4 shows the XRD patterns of Prussian blue and Prussian blue-coated PDDA@Iron oxide. Prussian blue displays a strong peak notably at 2è = 35.3°. The XRD patterns of Prussian blue with iron oxide showed a strong peak around 2è = 66.4° and 68.4°, respectively. All peaks are in good agreement both in their positions and relative intensities.

### 2.2. Removal of Radioactive Cesium

**Figure 5:**
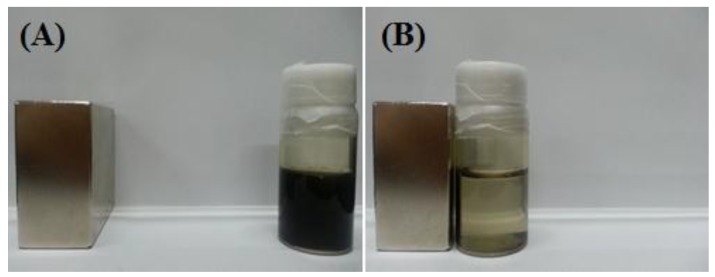
The separation of radioactive cesium (^137^Cs) using a magnet from Prussian blue-coated PDDA@Iron oxide.

The Prussian blue-coated PDDA@Iron oxide could be easily separated from water using an external magnet (Figure 5).

**Figure 6:**
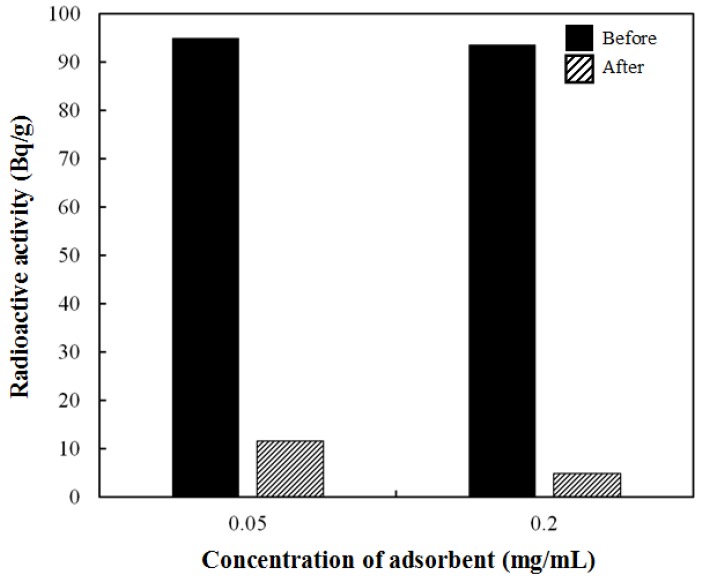
Removal efficiency of the radioactive cesium (^137^Cs) using Prussian blue-coated PDDA@Iron oxide.

Figure 6 shows that the Prussian blue-coated PDDA@Iron oxide possesses an excellent removal efficiency of the cesium (^137^Cs), more than 94% at a 0.2-mg/mL concentration. This means the potential adsorption of radioactive cesium due to the presence of Prussian blue-coated PDDA@Iron oxide. Taken together, these data reveal that the positively-charged surface of PDDA@Iron oxide nanoparticles is coated with negatively-charged Prussian blue. Eventually, Prussian blue displayed magnetism originating from the iron oxide core, and this characteristic would be suitable for the magnetically controlled elimination of radioactive cesium. Finally, this adsorbent showed high removal efficiency with respect to radioactive cesium. Therefore, Prussian blue-coated PDDA@Iron oxide demonstrated good potential for the treatment of radioactive cesium-contaminated water.

## 3. Experimental Section

### 3.1. Chemicals

All chemicals used in this work were of analytical reagent grade. Deionized water was used throughout the study. Iron(III) chloride hexahydrate (FeCl_3_·6H_2_O), iron(II) chloride tetrahydrate (FeCl_2_·4H_2_O), potassium hexacyanoferrate(II) trihydrate (K_3_[Fe(CN)_6_]·3H_2_O), poly(diallyldimethylammonium chloride) (PDDA) solution (20 wt%, MW 400,000~500,000), ammonium hydroxide (NH_4_OH) and hydrochloric acid (HCl) were purchased from Sigma-Aldrich Co., Ltd (St. Louis, MO, USA). Additionally, radioactive cesium chloride (^137^Cs) was obtained from the Korea Atomic Energy Research Institute (KAERI).

### 3.2. Preparation of Prussian Blue Coated-PDDA@Iron Oxide Nanoparticles

A solution (20 mL) containing FeCl_3_·6H_2_O (2.16 g), FeCl_2_·4H_2_O (0.8 g) and PDDA (1.0%, *v*/*v*) was deoxygenated by bubbling with nitrogen gas for 10 min, followed by heating to 80 °C. Subsequently, NH_4_OH (28%, 10 mL) was added rapidly to the heated solution, which was left to stir for another 1 h. After cooling to room temperature, the formed PDDA-Fe_3_O_4_ NPs were isolated with the help of a magnetic field and washed three times with deionized water. Additionally, these were dried at room temperature. Finally, purified PDDA-coated iron oxide nanoparticles (PDDA@Iron oxide) were obtained. PDDA@Iron oxide (0.1 g) was dispersed in 10 mL of DW, and 0.01 M HCl were added to adjust the pH to 6.0. Prussian blue (0.3 g), the pH of which ranges from 2.4 to 2.7, was dispersed in 10 mL of DW, to which 0.01 M NaOH was added to adjust the pH to 6.0. The slurry obtained was then mixed thoroughly at room temperature in a 50-mL polypropylene tube. DW (25 mL) was added to this mixture, and a permanent magnet (1.4 tesla) was used to eliminate any excess Prussian blue, which alone is not attracted by a magnetic field; so, we could magnetically separate the Prussian blue from the mixture of Prussian blue and non-magnetic Prussian blue.

### 3.3. Characterization and Adsorption Test

The crystal structure of the nanoparticles was characterized by XRD spectroscopy (Bruker D2 phaser, Rheinstetten, Germany). FTIR spectra of the nanoparticles were recorded using a Thermo Scientific Nicolet iS5 (Madison, WI, USA). The particle size and morphology of the nanoparticles were investigated using a TEM at an accelerating voltage of 300 keV (Tecnai G2 F30, Hillsboro, OR, USA). The nanoparticle sizes were determined by a dynamic light scattering (DLS) device (Malvern Zetasizer nano-ZS90, Malvern, UK). The ability of the Prussian blue-coated PDDA@Iron Oxide nanoparticles to adsorb radioactive cesium (^137^Cs) was investigated in an aqueous solution. Two amounts of the Prussian blue coated-PDDA@Iron oxide nanoparticles (0.5, 2 mg) were mixed with 10 mL of the aqueous solution of the radioactive cesium for 3 h on a shaker to reach the adsorption equilibrium. The nanoparticles were removed from the solution using magnetic decantation. The concentrations of the radioactive cesium (^137^Cs) before and after treatment with the Prussian blue-coated PDDA@Iron oxide nanoparticles were measured using a High-Purity Germanium (HPGe) detector (Canberra, Meriden, CT, USA).

## 4. Conclusions

In summary, we elucidated the ability of Prussian blue-coated magnetic nanoparticles to eliminate radioactive cesium, as in radioactive contaminated water. The magnetic Prussian blue nanoparticles were synthesized and characterized for their physical and radioactive cesium adsorption properties. Additionally, this adsorbent showed high removal efficiency with respect to radioactive cesium. Furthermore, the magnetic adsorbent with Prussian blue possesses high cesium adsorption capacity and can be easily recovered once spread into an open environment.
